# SLEEP QUALITY AND DEPRESSIVE SYMPTOMS IN LATER-LIFE: CROSS-SECTIONAL EXAMINATION OF COGNITIVE MECHANISMS

**DOI:** 10.1093/geroni/igz038.3198

**Published:** 2019-11-08

**Authors:** David Brush, Daniel Paulson, Manuel Herrera Legon, Nicholas James, Jennifer Scheurich, Brittany Stevenson

**Affiliations:** University of Central Florida, Orlando, Florida, United States

## Abstract

Sleep quality relates to depressive symptom endorsement. The mechanisms relating these variables are not clearly elucidated, though inhibitory control and rumination are believed to play key roles. The current study aims to elucidate the relationship between sleep quality and depressive symptoms by examining the moderated mediating effect of inhibitory control and rumination. The sample included 41 community-dwelling older adults (age 70 and older). Measures included the Pittsburg Sleep Quality Inventory, a Stroop task (inhibitory control), the Ruminative Responses Scale, and the Geriatric Depression Scale. A series of bootstrapped models were employed to test hypotheses using a stepped approach. Poorer sleep quality was associated with higher rumination and depressive symptoms; however, these associations were no longer significant among older adults with higher inhibitory control. The association between sleep quality and depression was fully attenuated by rumination, and inhibitory control significantly moderated the association between sleep quality and rumination in the final model. Among community-dwelling older adults, the association between sleep quality and depression is mediated by rumination, and this effect is mitigated by inhibitory control. As such, these findings suggest that inhibitory control may be a relevant target for intervention in older adults with poor sleep quality, rumination, and depressive symptoms.

